# Statistical description of the denatured structure of a single protein, staphylococcal nuclease, by FRET analysis

**DOI:** 10.1007/s12551-017-0334-y

**Published:** 2017-11-25

**Authors:** Mariko Yamaguchi, Emi Ohta, Takuya Muto, Takayoshi Watanabe, Takahiro Hohsaka, Yoichi Yamazaki, Hironari Kamikubo, Mikio Kataoka

**Affiliations:** 1 Graduate School of Materials Science, Nara Institute of Science and Technology, Ikoma, Nara, 630-0192 Japan; 2 Present Address: IMRA America, Inc., 48834 Kato Road, Fremont, CA 94538 USA; 3 School of Materials Science, Japan Advanced Institute of Science and Technology, Nomi, Ishikawa 923-1292 Japan; 4 Present Address: Research Center for Neutron Science and Technology, Comprehensive Research Organization for Science and Society (CROSS), 162-1 Shirakata, Tokai, Ibaraki, 319-1106 Japan

**Keywords:** Protein, Denatured structure, FRET, Random coil, Staphylococcal nuclease

## Abstract

Structural characterization of fully unfolded proteins is essential for understanding not only protein-folding mechanisms, but also the structures of intrinsically disordered proteins. Because an unfolded protein can assume all possible conformations, statistical descriptions of its structure are most appropriate. For this purpose, we applied Förster resonance energy transfer (FRET) analysis to fully unfolded staphylococcal nuclease. Artificial amino acids labeled with a FRET donor or acceptor were introduced by an amber codon and a four-base codon respectively. Eight double-labeled proteins were prepared, purified, and subjected to FRET analysis in 6 M urea. The observed behavior could be explained by a power law, *R* = *αN*^0.44^, where *R*, and *N* are the distance and the number of residues between donor and acceptor, and *α* is a coefficient. The index was smaller than the value expected for an excluded-volume random coil, 0.588, indicating that the fully unfolded proteins were more compact than polypeptides in good solvent. The FRET efficiency in the native state did not necessarily correlate to the distance obtained from crystal structure, suggesting that other factors such as the orientation factor made a substantial contribution to FRET.

## Introduction

Protein stability and folding are among the most important topics in protein science. Many excellent studies have been published on this subject, including the pioneering work of Anfinsen, who was awarded the Nobel Prize in Chemistry 1972 (Anfinsen 1972). The folded structure of a protein is located at the minimum of the free-energy potential surface. Hence, folding is considered as a potential-minimum search process starting at a high-energy state (Levinthal 1968; Dill 1993; Onuchic et al. 1995; Dill and Chan 1997). Recent advances in structural and computational biology have provided deep insight into the stabilities and folding mechanisms of proteins. The atomic structures of various proteins have been solved to understand necessary atomic interaction for folding. The solved structure is the destination of folding. On the other hand, detailed structural information about denatured or unfolded structures, the initial point of folding, remains insufficient.

The importance of information on denatured structures began to be emphasized in the 1990’s (Dill and Shortle 1991). The denatured structure is considered as the initial state in the process of protein-folding. Exploration of the folding mechanism by simulation is based on the idea that the initial state can assume all possible conformations, i.e., that it is a random coil (Pappu et al. 2000), but it remains to be resolved whether this is truly the case. On one hand, the scaling of the hydrodynamic radius as a function of molecular weight (chain length) indicates that the denatured state exhibits random coil behavior (Tanford et al. 1966; Kohn et al. 2004). On the other hand, NMR studies have shown that several residual structures remain in the denatured state (Neri et al. 1992; Kazmirski et al. 2001; Shortle and Ackerman 2001), and mutations affect both denatured and folded structures (Shortle and Meeker 1986); together, these observations suggest that the denatured structure is not necessarily a random coil. The apparent discrepancy may arise from the fact that the former studies were based on a statistical description of various proteins, whereas the latter were based on local structural information for a single protein. Accordingly, a statistical description of the structure of a single protein molecule in the denatured state would reconcile the discrepancy. To this end, we investigated the pairwise distances between residues over the entire polypeptide chain in a single protein, staphylococcal nuclease (SNase), using Förster resonance energy transfer (FRET).

To conduct FRET structure analysis, we need to introduce both donor and acceptor fluorescent dyes into a protein. For this purpose, a standard method is labeling of a reactive residue, such as cysteine or lysine. However, specific double-labeling at the desired positions and purification of properly labeled proteins are challenging, and it is not straightforward to prepare several sets of proteins double-labeled at different positions. To overcome these difficulties, we applied a labeling method that uses the amber codon and an artificial four-base codon (Hohsaka et al. 1999), corresponding to the donor BODIPY FL amino phenylalanine and acceptor BODIPY 558 amino phenylalanine (Kajihara et al. 2006) respectively. The donor site is designated by the amber codon, and the acceptor site is fixed at the fifth amino acid position by the four-base codon, CGGG. At the C-terminal end, the 6-histidine tag is introduced to facilitate purification. A total of eight double-labeled proteins were prepared and purified. The donor site of each protein was located at residue 33, 48, 70, 97, 123, 134, 143, or 146; accordingly, the double-labeled proteins are represented as, e.g., 33_D_5_A_ and 48_D_5_A_.

Measurement of fluorescence spectra and derivation of FRET efficiency were successful for all labeled SNases with and without 6 M urea. The influence of urea on the fluorescence of donor and acceptor dyes was negligible compared to the FRET between the dyes introduced into a single SNase molecule. Complete unfolding of SNase at 6 M urea was confirmed by CD spectra. The FRET efficiency at 6 M urea showed that the *R*–*N* relationship obeys a power law with an index of 0.44, where *R* and *N* are the distance and the number of residues between donor and acceptor. This is smaller than the index of an excluded-volume random coil, 0.588 (De Gennes 1979; Kohn et al. 2004). The statistical description of the unfolded structure of a single protein suggests that the unfolded structure cannot be described by well-known random-coil polymer statistics, but instead is more compact. This is the first statistical description of the denatured structure of a single protein. Our findings indicate that FRET analysis of a single polypeptide is both effective and promising.

## Materials and methods

### Design of DNA sequence

**Fig. 1:**
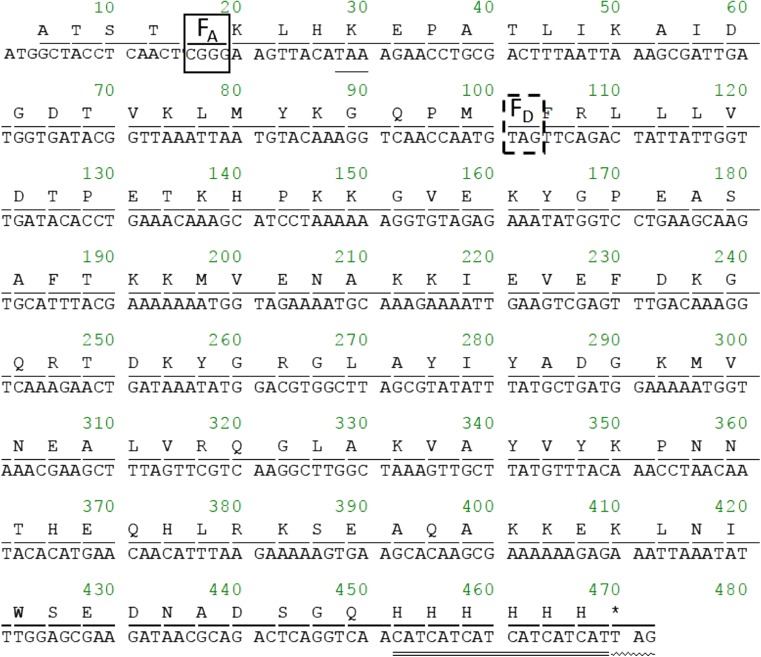
Designed DNA sequence for the acceptor site at the fifth position and the donor site at the 33rd position (33_D_5_A_). The four-base codon and amber codon are demarcated by a *solid box* and a *broken box* respectively. The *single underline* represents the expected stop codon when the four-base codon is read as an ordinary three-base codon and a frameshift occurs. The *double underline* represents the 6xHis-tag. The stop codon, indicated by the *wavy underline*, is TGA

Figure 1 shows the DNA sequence for the protein with the acceptor site at the fifth position and the donor site at the 33rd position (33_D_5_A_). When the four-base codon, CGGG, is translated into CGG as a normal three-base codon, a frameshift occurs, and a polypeptide with a completely different amino acid sequence is generated. To avoid such misreading, the expected stop codon (TAA) appears immediately. This strategy was also used for the other seven polypeptides. The donor site is designated by the amber codon, TAG. At the C-terminal end, the 6-histidine tag is added for purification. To avoid introducing another donor, the stop codon used in this construct is TGA instead of TAG. Only the successfully translated polypeptide can be purified on Ni-chelate beads. Two single-labeled proteins with either the four-base codon or the amber codon at the fifth position (5_D_ or 5_A_ respectively) were also prepared.

### Protein expression and purification

Aminoacyl tRNA of BODIPY-phenylalanine with the anti-codon for the four-base codon or amber codon was synthesized in each experimental run using a previously described chemical aminoacylation method (Kajihara et al. 2006; Iijima and Hohsaka 2009). Proteins were expressed using a cell-free expression system containing the aminoacyl tRNA and relevant mRNA, as previously described (Iijima and Hohsaka 2009). The volume of each cell-free reaction was 100 μl. Full-length double-labeled proteins were purified on Ni-NTA beads (MagneHis Ni-particles, Promega, Madison, WI, USA) and desalted using a Zeba desalting column (Thermo Fisher Scientific, Waltham, MA, USA). The proteins were eluted in fluorescence measurement buffer (30 mM sodium phosphate, 300 mM NaCl, 0.005% Brij-35, 0.1% PEG-8000, pH 7.8).

Expression and purification were monitored by fluorescence imaging of PAGE gels and western blotting. The gel pattern was recorded on an LAS4000 (GE Healthcare) and an FMBIO-III (Hitachi Software Engineering).

### Measurement of fluorescence

Fluorescence spectra were measured on an F-2500 fluorescence spectrophotometer (Hitachi) immediately after purification to prevent chromophores from degrading. After preparation of a 100 μl solution either with or without 6 M urea, two fluorescence spectra were measured with sequential excitation at 490 nm and 530 nm. The ratio of the fluorescence intensity at 570 nm to that at 490 nm, *RI*, was obtained from the fluorescence spectra with 490 nm excitation for further analysis.

### Analysis of fluorescence spectra

FRET efficiency, *E*, is expressed as

1 $$ E=\frac{1}{1+{\left(\frac{R}{R_0}\right)}^6}. $$

*R*_0_ is the Förster distance, at which the FRET efficiency is 0.5, expressed as

2 $$ {R}_0^6=\frac{9{Q}_0\left(\ln 10\right){\kappa}^2J}{128{\pi}^5{n}^4{N}_A}, $$

where *Q*_0_, *J*, *κ*^2^, *n*, and *N*_A_ are the quantum yield of donor, the overlap integral of the fluorescence spectrum of the donor and the absorption spectrum of the acceptor, the orientation factor, the refractive index of medium, and Avogadro’s number respectively (Valeur 2001). In the present case, the Förster distance was estimated to be 59.6 Å (Iijima and Hohsaka 2009).

Experimentally, *E* is estimated from *RI*. To derive the relationship between *E* and *RI*, we first define the fluorescence spectrum of a double-labeled protein *I*_DA_ as follows:

3 $$ {I}_{\mathrm{D}\mathrm{A}}={C}_{\mathrm{D}}{I}_{\mathrm{D}}+{C}_{\mathrm{A}}{I}_{\mathrm{A}}, $$

where *I*_D_ and *I*_A_ are the normalized fluorescence spectra of donor and acceptor respectively. The fluorescence spectrum of a single-labeled protein, 5_D_ (5_A_), was used to obtain *I*_D_ (*I*_A_). *C*_D_ and *C*_A_ are the fluorescence intensities of donor and acceptor respectively. From Eq. (3), *RI* can be expressed in terms of *C*_D_ and *C*_A_ as

4 $$ RI=\frac{C_{\mathrm{A}}}{C_{\mathrm{D}}}. $$

*C*_A_ and *C*_D_ are defined by Eq. (5):

5 $$ {\displaystyle \begin{array}{l}{C}_{\mathrm{A}}={\varepsilon}_{\mathrm{D}}(490)E{\phi}_{\mathrm{A}}(570)+{\varepsilon}_{\mathrm{A}}(490){\phi}_{\mathrm{A}}(570)\\ {}{C}_{\mathrm{D}}={\varepsilon}_{\mathrm{D}}(490)\left(1-E\right){\phi}_{\mathrm{D}}(512)\end{array}}, $$

where *ε*_D_(490), *ε*_A_(490), *ϕ*_D_(512), and *ϕ*_A_(570) are the absorption coefficient at 490 nm of the donor, the absorption coefficient at 490 nm of the acceptor, the fluorescence quantum yield at 512 nm of the donor, and the fluorescence quantum yield of the acceptor at 570 nm respectively. Consequently, *E* can be expressed in terms of *RI* as

6 $$ E(R)=\frac{RI(R)- RI\left(R=\infty \right)}{RI(R)+ RI\left(R=\infty \right)\left(\frac{\varepsilon_{\mathrm{D}}(490)}{\varepsilon_{\mathrm{A}}(490)}\right)}, $$

where *RI*(*R* = ∞) is the ratio of emission peak of the acceptor to that of the donor without FRET, expressed as

7 $$ RI\left(R=\infty \right)=\frac{\varepsilon_{\mathrm{A}}(490){\phi}_{\mathrm{A}}(570)}{\varepsilon_{\mathrm{D}}(490){\phi}_{\mathrm{D}}(512)}. $$

## Results and discussion

### Purification of double-labeled proteins

**Fig. 2:**
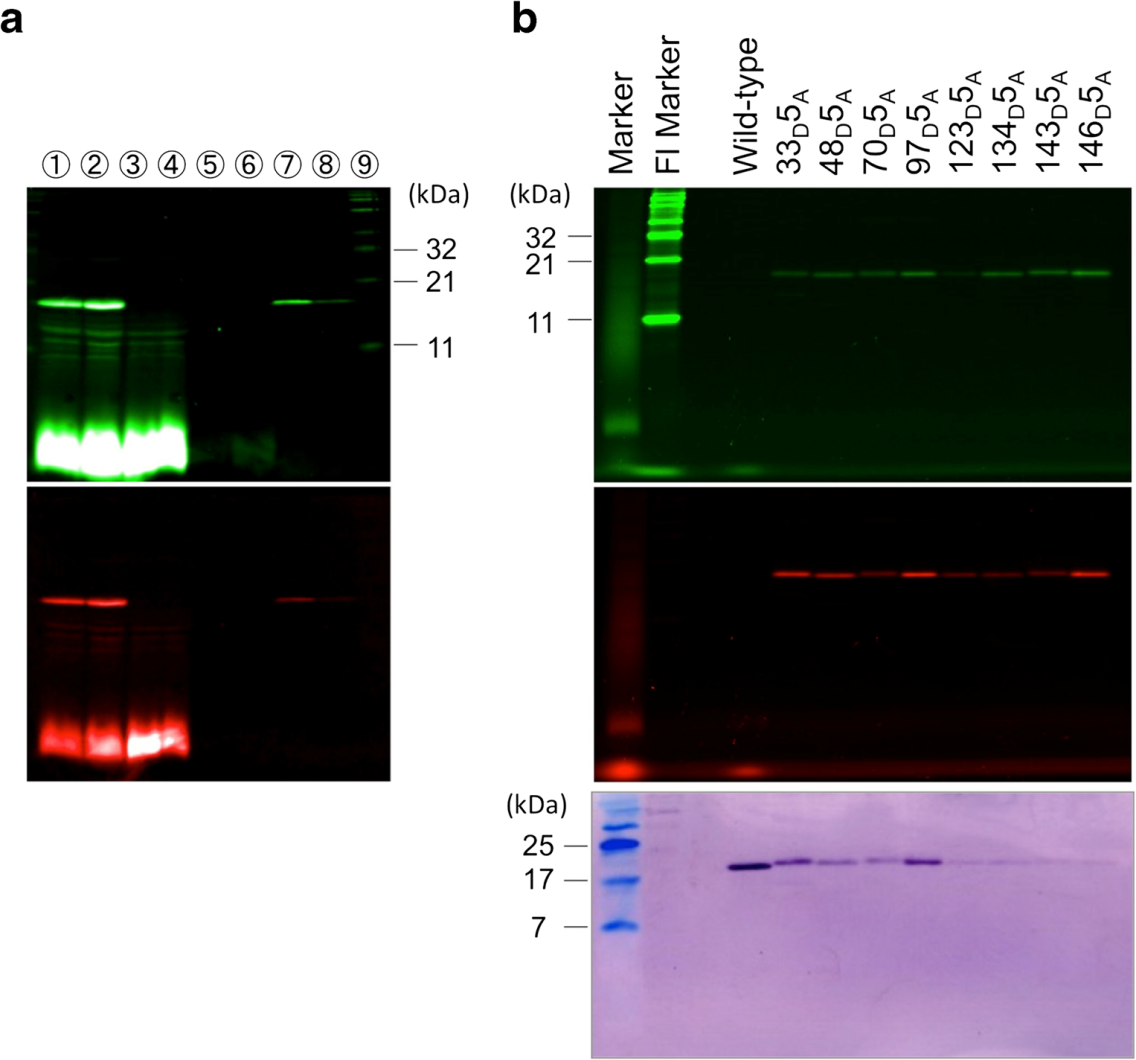
**a** Gel images of the purification process of 33_D_5_A_. Lanes are as follows: *1* and *2*, cell-free translation solution; *3* and *4*, supernatant of Ni-beads; *5* and *6*, cleaning solution of Ni-beads; *7*, elution of Ni-beads; *8*, final elution by gel filtration chromatography; *9*, fluorescent marker. *Top*, fluorescence image of 515 nm emission at 460 nm excitation; *bottom*, fluorescence image of 605 nm emission at 460 nm excitation. Images were taken on an LAS4000 (GE Healthcare). **b** Gel images of eight purified samples. *Top*, fluorescence image of 520 nm emission at 488 nm excitation; *center*, fluorescence image of 580 nm emission at 532 nm excitation. Images were taken on an FMBIO-III (Hitachi Software Engineering). *Bottom*, western blot analysis of double-labeled His-tagged SNase. Proteins were visualized with an anti–His-tag antibody

Figure 2(a) shows an example of the purification process of products obtained using the cell-free translation system, monitored by fluorescence detection of the PAGE pattern of protein 33_D_5_A_. The products consist of the properly double-labeled protein; polypeptide with the acceptor, whose translation is stopped at the amber codon (acceptor single-labeled polypeptide); and the short polypeptide without any dye. The sharp strong band around 17 kD corresponds to the double-labeled protein. There are other bands that were not identified, but might have originated from truncated double-labeled polypeptides. The strong background of the gel front consists of aminoacyl tRNA with BODIPY phenylalanine and liberated BODIPY phenylalanine. Both the unexpected bands and strong background appeared in the supernatant of Ni-beads. The final eluted sample contained only the double-labeled full-length SNase. Further purification was carried out by gel-filtration column chromatography. The eight double-labeled proteins were successfully purified, as shown in Fig. 2(b).

### Fluorescence spectra

Fluorescence measurements to evaluate the ratio of the fluorescence intensity from the acceptor to the donor, were repeated several times to assess the degradation of the chromophores. The fluorescence intensity gradually decreased with the number of measurements; the decrease in fluorescence intensity was smaller in 6 M urea than under physiological conditions. The intensity decreased by ~10% after five measurements: the spectral shape changed very little over the course of the first three measurements, but began to change slightly starting at the fourth measurement. Based on this observation, we concluded that the degradation of the chromophores was negligible for up to three measurements. Consequently, we used the ratio of the fluorescence intensity (*RI*) for the purposes of evaluation.

**Fig. 3:**
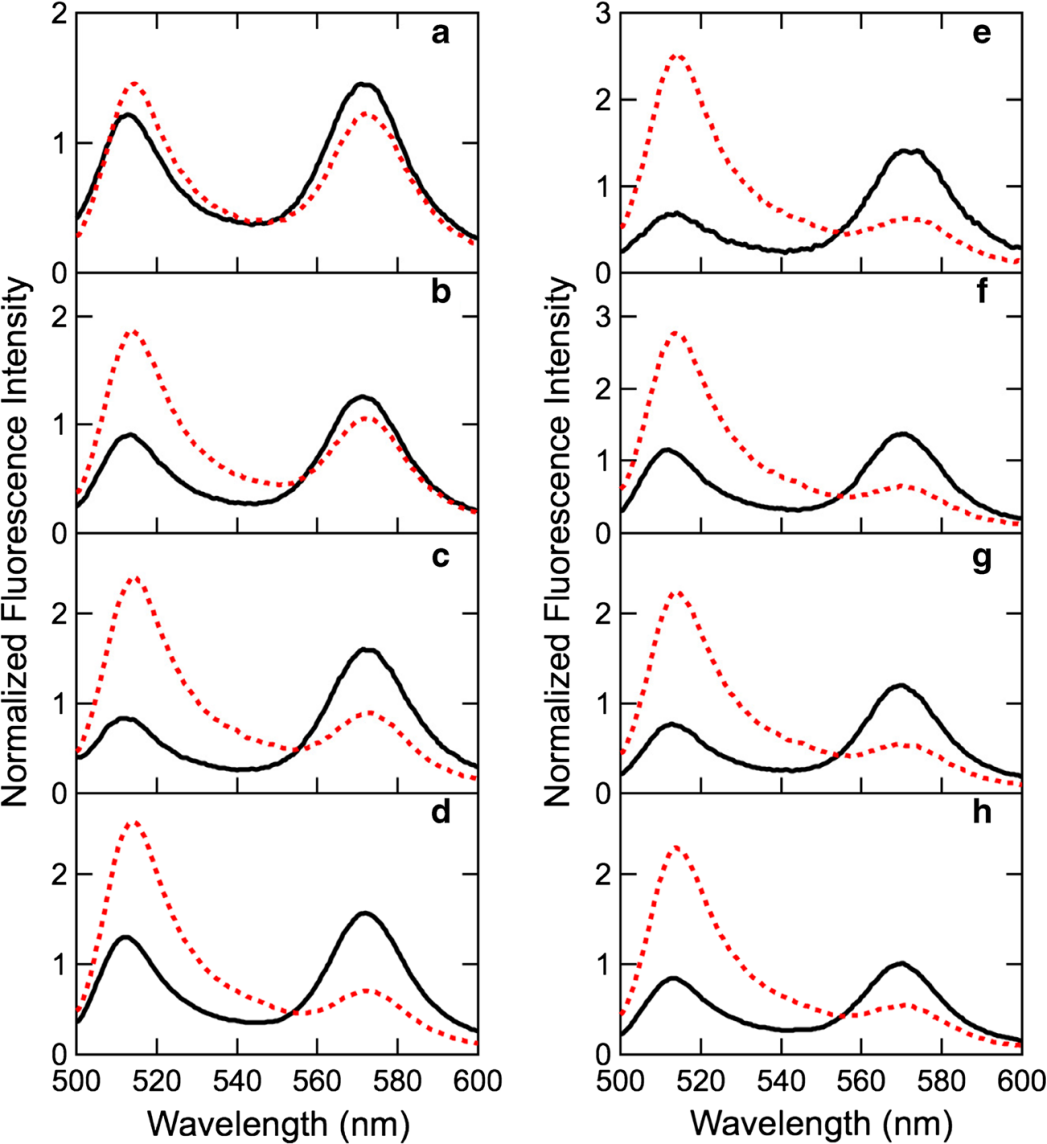
Normalized fluorescence spectra of double-labeled SNase in the native state (*black solid line*) and in the presence of 6 M urea (*red broken line*). **a** 33_D_5_A_, **b** 48_D_5_A_, **c** 70_D_5_A_, **d** 97_D_5_A_, **e** 123_D_5_A_, **f** 134_D_5_A_, **g** 143_D_5_A_, and **h** 146_D_5_A_

Figure 3 shows fluorescence spectra of eight double-labeled SNases in the presence or absence of 6 M urea with excitation at 490 nm. Two peaks are clearly observed in all spectra. The emission peak at 512 nm is attributed to fluorescence from the donor, whereas the peak at 570 nm is attributed to fluorescence from the acceptor. Only the 570 nm peak was observed when 530 nm light was used for excitation (data not shown). Because the emission at 570 nm with 530 nm excitation results purely from the direct excitation of the acceptor and does not reflect the contribution of FRET, the fluorescence intensity is proportional to the concentration of the acceptor, i.e., the labeled protein. Therefore, we normalized the fluorescence spectra in Fig. 3 against the fluorescence intensity at 570 nm with 530 nm excitation so that the normalized fluorescence intensity would be independent of protein concentration. The fluorescence intensity at 570 nm with 530 nm excitation did not change upon addition of 6 M urea, which enables us to compare the normalized fluorescence spectra between the presence and absence of urea.

Upon urea denaturation, the emission peak from the acceptor decreased, whereas the peak from the donor increased, indicating that the FRET efficiency of the native state is higher than that of the unfolded state. Thus, the fluorescence spectra are able to probe the structural change due to denaturation. The spectral shapes differed between the labeled sites in both the native and the unfolded states. This site-dependent FRET could provide structural information about the two states.

FRET analyses for native structures were conducted for the fluorescence spectra in Fig. 3. Each spectrum was reconstituted from donor and acceptor fluorescence spectra to obtain *C*_D_ and *C*_A_ in Eq. (3). Then, *RI* was obtained from Eq. (4), and *E* was obtained from Eq. (6). The *RI*(*R* = ∞) in Eq. (6) is 0.072 ± 0.001, which was experimentally obtained by measuring a fluorescence spectrum of a dilute solution of a 1:1 mixture of BODIPY 558 phenylalanine and BODIPY FL phenylalanine. Furthermore, for the native state, the FRET distance between the donor and acceptor *R* was estimated from Eq. (1). The obtained *RI*, *E*, and *R* values are summarized in Table 1.

**Table 1:** Quantities obtained by FRET analysis

Protein	Native state				6 M urea	
	*RI*	*E*	*R* (Å)	*R*_ca_* (Å)	*RI*	*E*
33_D_5_A_	1.17 (0.02)**	0.78 (0.03)	48 (1)	26	0.81 (0.01)	0.71 (0.04)
48_D_5_A_	1.38 (0.02)	0.81 (0.03)	47 (1)	42	0.524 (0.006)	0.60 (0.05)
70_D_5_A_	1.97 (0.02)	0.86 (0.02)	44 (1)	25	0.326 (0.002)	0.46 (0.06)
97_D_5_A_	1.179 (0.005)	0.78 (0.03)	48 (1)	20	0.233 (0.005)	0.35 (0.06)
123_D_5_A_	2.23 (0.03)	0.88 (0.02)	43 (1)	20	0.202 (0.002)	0.30 (0.06)
134_D_5_A_	1.166 (0.006)	0.78 (0.03)	48 (1)	34	0.177 (0.002)	0.26 (0.06)
143_D_5_A_	1.525 (0.008)	0.83 (0.03)	46 (1)	(39)	0.190 (0.002)	0.28 (0.06)
146_D_5_A_	1.141 (0.007)	0.78 (0.03)	48 (1)	(39)	0.182 (0.002)	0.27 (0.06)

*R_ca_ is given by the distance between Cα atoms. Note that the coordinates of the 143rd and 146th positions are ambiguous (given by parentheses), as is the fifth position. Because the fifth position is common to all proteins, the values can describe the qualitative relationship

**Estimated errors are given in parentheses following the corresponding values

### Site-dependence of FRET efficiency

**Fig. 4:**
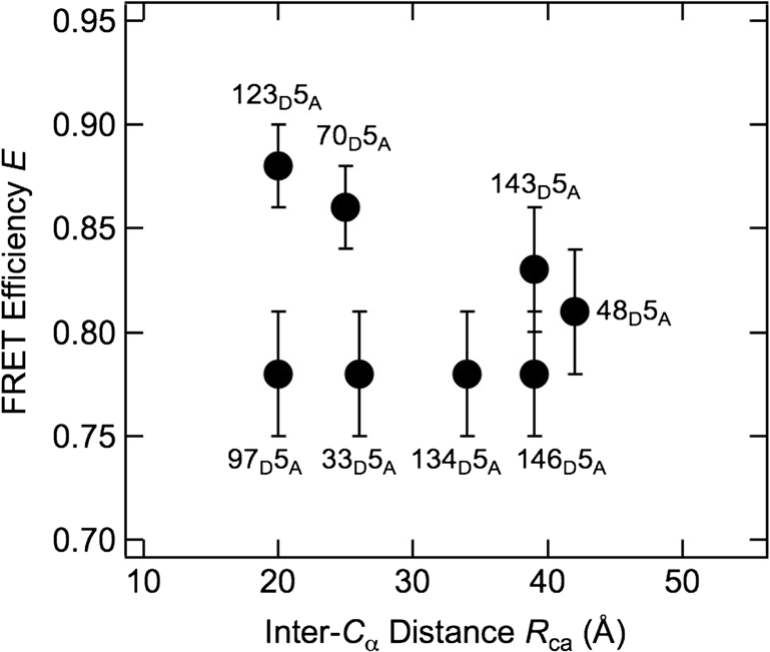
Comparison of the distance estimated by FRET with the inter-Cα distance obtained from the coordinates in 2SNS (Cotton et al. 1979). Note that the coordinates of the 143rd and 146th position are ambiguous. Although the coordinates of the fifth position are also ambiguous, we used as a reference point because this residue is common for all double-labeled proteins

The FRET efficiency *E* is plotted as a function of the inter-Cα distance, *R*_ca_, for the native state in Fig. 4. *R*_ca_ was obtained from the crystal structure of SNase (PDB code: 2SNS) (Cotton et al. 1979) and is shown in Table 1. It is difficult to find the Cα atom coordinate of the fifth position in the available crystal structural data because the five N-terminal residues and eight C-terminal residues fluctuate even in the native state. We referred to 2SNS, in which the coordinates of main chain atoms of these residues are specified with a weak electron density distribution.

If *E* depends only on *R*_ca_ as in Eq. (1), *R* should be proportional to *R*_ca_. In fact, *R* for 48_D_5_A_, 70_D_5_A_, and 123_D_5_A_ were proportional to *R*_ca_, as shown in the *R*–*R*_ca_ plot in Fig. 4. However, *R* for 33_D_5_A_, 97_D_5_A_, and 134_D_5_A_ were independent of *R*_ca_, indicating that *R* obtained from *E* by Eq. (1) is not necessarily reflected in the native structure. Two sets of data, from 143_D_5_A_ and 146_D_5_A_, were excluded from this discussion because these *R*_ca_s are suspicious from the crystal structure data. Comparison of the position of the donor site between the groups revealed that the former residues are located on long loops, whereas the latter residues are on secondary structural elements. Flexibility of residues may be responsible for these differences in the *R–R*_ca_ relationship.

Figure 4 suggests that several factors affect *E*. One of these factors could be the orientation factor *κ*^2^ in Eq. (2), which can have a value between 0 and 4 depending on the orientation (Valeur 2001). In solution, it is usually set at 2/3, under the assumption that the chromophores can take all possible orientations. In the native state, however, the residues are tightly packed and therefore have restricted rotational motion. The effect of the orientation factor could be the origin of the discrepancy between *R*_ca_ and *R* derived from *E*. These observations indicate that labeling at an improper site could yield incorrect structural information.

**Fig. 5:**
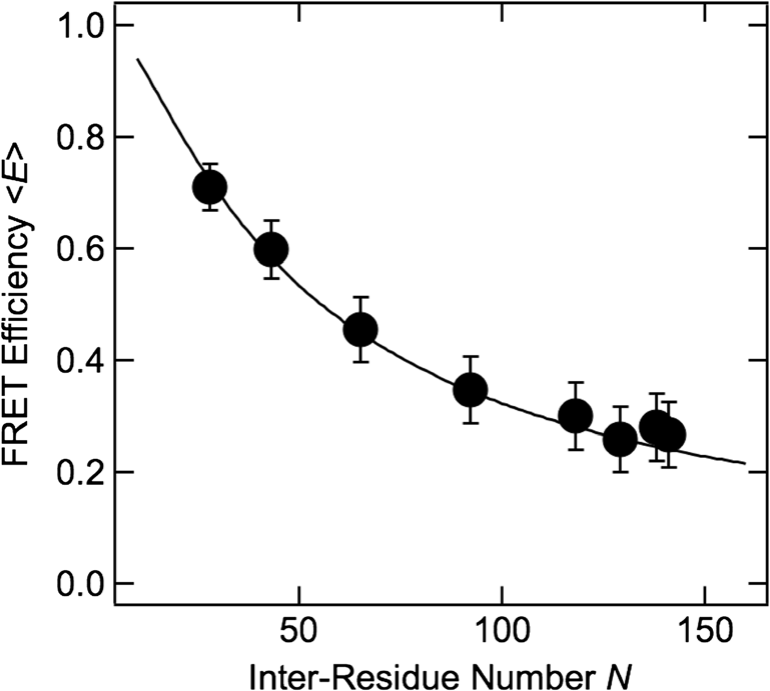
FRET efficiency of the fully unfolded state in 6 M urea, plotted as a function of inter-residue number. The *solid line* is the result of fitting, described in the text

Figure 5 shows the dependence of *E* on the inter-residue number, *N*, in the fully unfolded state (i.e., in the presence of 6 M urea). *E* decreased monotonously as *N* increased. To describe the statistical properties of the fully unfolded state of SNase, the *E*–*N* relationship can be analyzed under an assumption of power-law dependence of *R* on *N*, as follows (De Gennes 1979):

8 $$ R=\alpha {N}^{\beta }. $$

Because the fully unfolded state assumes many conformations, *R* cannot be determined as a discrete value, but should instead be represented by a distribution function. In that case, *E* is defined by Eq. (9) instead of Eq. (1).

9 $$ \left\langle E\right\rangle =\int p(R) EdR=\int \frac{p(R)}{1+{\left(\frac{R}{R_0}\right)}^6} dR, $$

where *p* is the distribution of *R.* Here we use the following distribution function,

10 $$ p(R)=4\pi {R}^2{\left(\frac{3}{2\pi \left\langle {R}^2\right\rangle}\right)}^{\frac{3}{2}}\exp \left(-\frac{3{R}^2}{2\left\langle {R}^2\right\rangle}\right). $$

Equation (10) is a three-dimensional Gaussian distribution that is often used in FRET analysis (Sherman and Haran 2006). We assume that the mean distance *R*_mean_ obeys the power law in Eq. (8), as follows:

11 $$ {R}_{\mathrm{mean}}=\alpha {N}^{\beta }. $$

The fitting of Eqs. (9)–(11) to the *E*–*N* plot in Fig. 5 gives *β* = 0.44 ± 0.01. The fitted line is shown in Fig. 5. When the distance distribution is not taken into account, *β* = 0.20 ± 0.01 is obtained. Theoretical studies have shown that *β* = 0.588 for an exclusive-volume random coil in good solvent (Kohn et al. 2004), and experimental studies have consistently shown that *β* = 0.588 as reflected by measurements of radius of gyration, intrinsic viscosity, etc. for proteins in high-concentration denaturant (Tanford et al. 1966; Kohn et al. 2004). The result obtained in this study, *β* = 0.44 ± 0.01, is smaller than previously reported results, although it becomes closer if the distance distribution is taken into account. Note also that the present result refers to a single polypeptide, whereas the previous results were obtained in various proteins. In the other words, the present result is sequence-dependent, whereas previous results were sequence-independent.

Physical quantities such as radius of gyration are probably equivalent to the end-to-end distance of a polymer chain because they all capture the whole molecule. On the other hand, the inter-residue distance probes a segment within a polymer chain. The distribution of a segment could differ from that of a full-length polymer chain, even when they are of the same length, because the conformations of the segment could be restricted by residues outside the segment. Thus, the statistical description of a segment might differ from that of the whole molecule.

The statistical description of various polymer chains could blend all of the unique characteristics together to yield a simple power-law behavior. In fact, *β* = 0.588 has been obtained even for proteins containing secondary structure (Fitzkee and Rose 2004). On the other hand, in this study, which examined a single molecule, we obtained *β* = 0.44. The different statistics observed for a single protein may extract information about unique structural features. Although persistent residual structure is not observed in Fig. 5, the fact that *β* = 0.44 suggests that unfolded SNase has a more compact structure than a random coil. In a previous study, a hydrophobic cluster was observed even in 7 M urea (Neri et al. 1992). Thus, the residue distribution of unfolded SNase might be confined due to hydrophobic collapse. In fact, polymers in poor solvent have *β* < 0.588; in this case, the interactions among segments are attractive due to the solvophobic effect. This observation is important and useful for understanding not only the structure of the unfolded state, but also the mechanism of protein folding. It also provides insight into the possible structures of intrinsically disordered proteins.

## Conclusion

We successfully introduced a fluorescent donor and acceptor into SNase using a four-base codon and an amber codon. Using FRET analysis, we carried out a systematic investigation of the distribution of the designated residues in the fully unfolded state. The introduction of artificial amino acids with fluorescent chromophores represents a unique and promising strategy for studying protein folding by FRET. Based on our results, we were able to provide, for the first time, the statistical description of the unfolded structure of a single polypeptide. The data revealed a scaling law distinct from that of a random coil, suggesting that the unfolded structure of SNase is more compact than random coil. On the other hand, distance determination between the donor and the acceptor in the native state is reasonable in some cases but not necessarily in others, depending on the donor position in comparison with the crystal structure. Therefore, the application of FRET analysis to the native state should be performed carefully.
